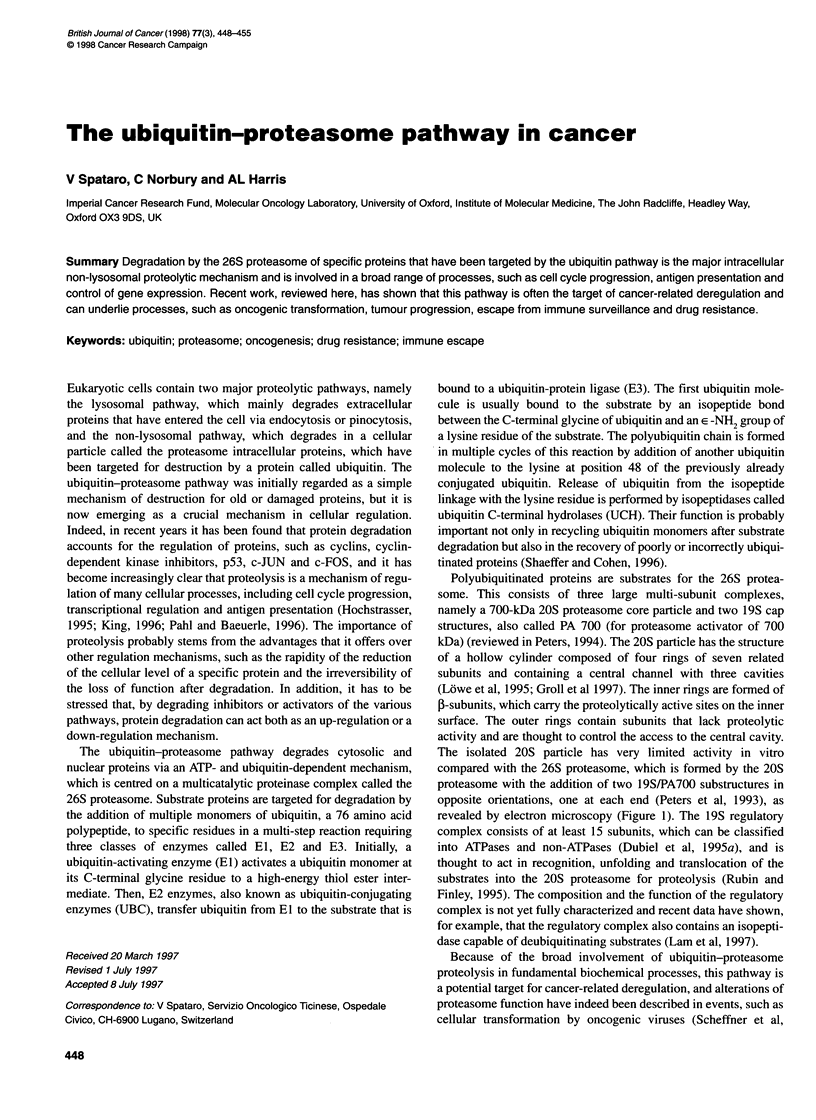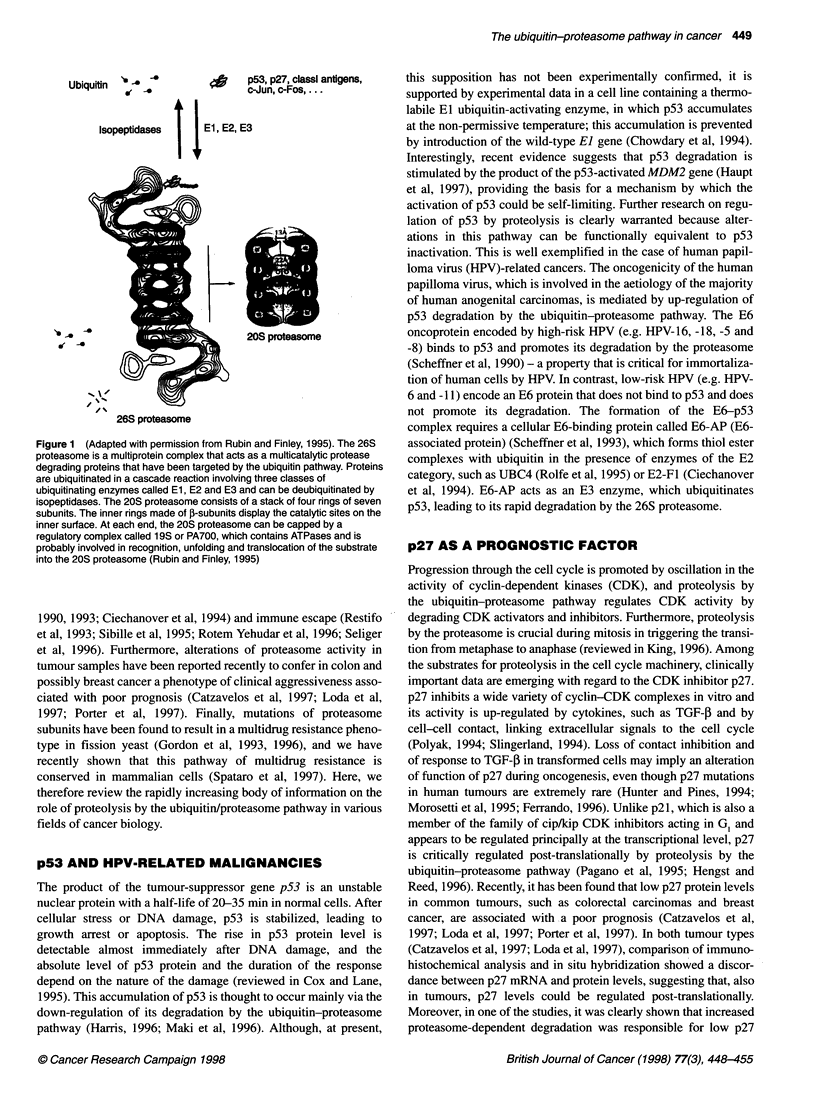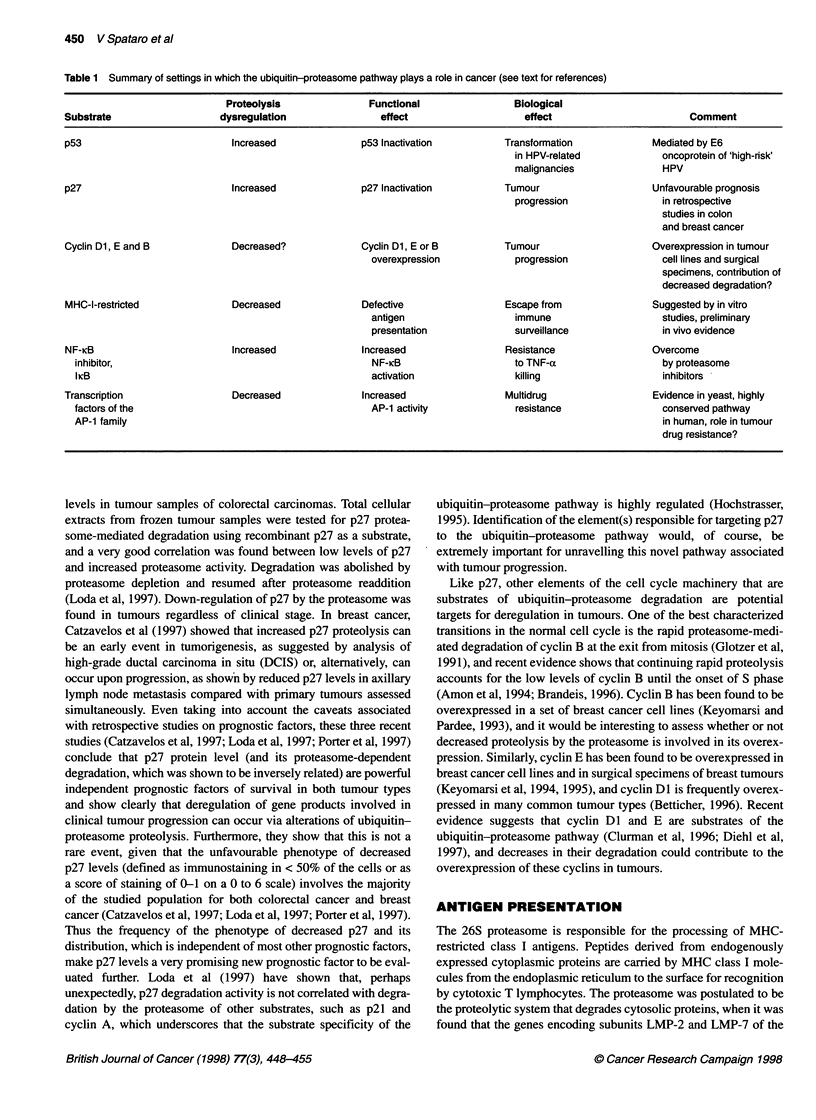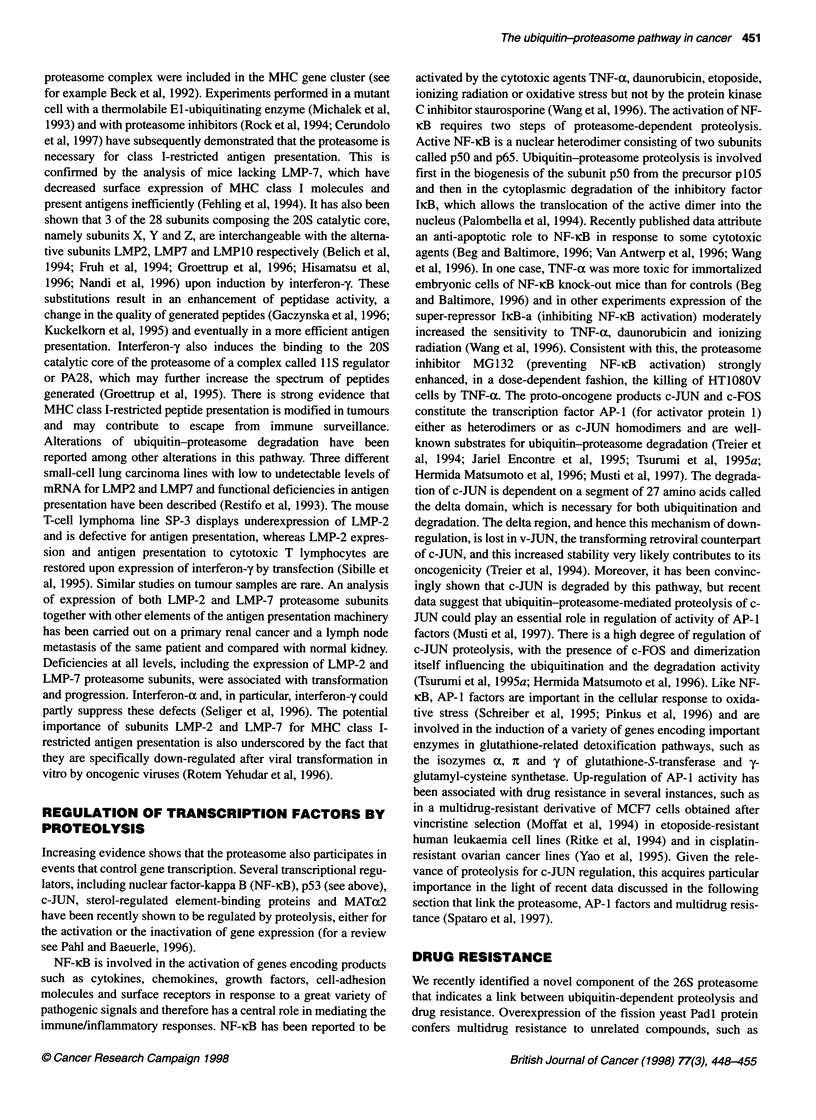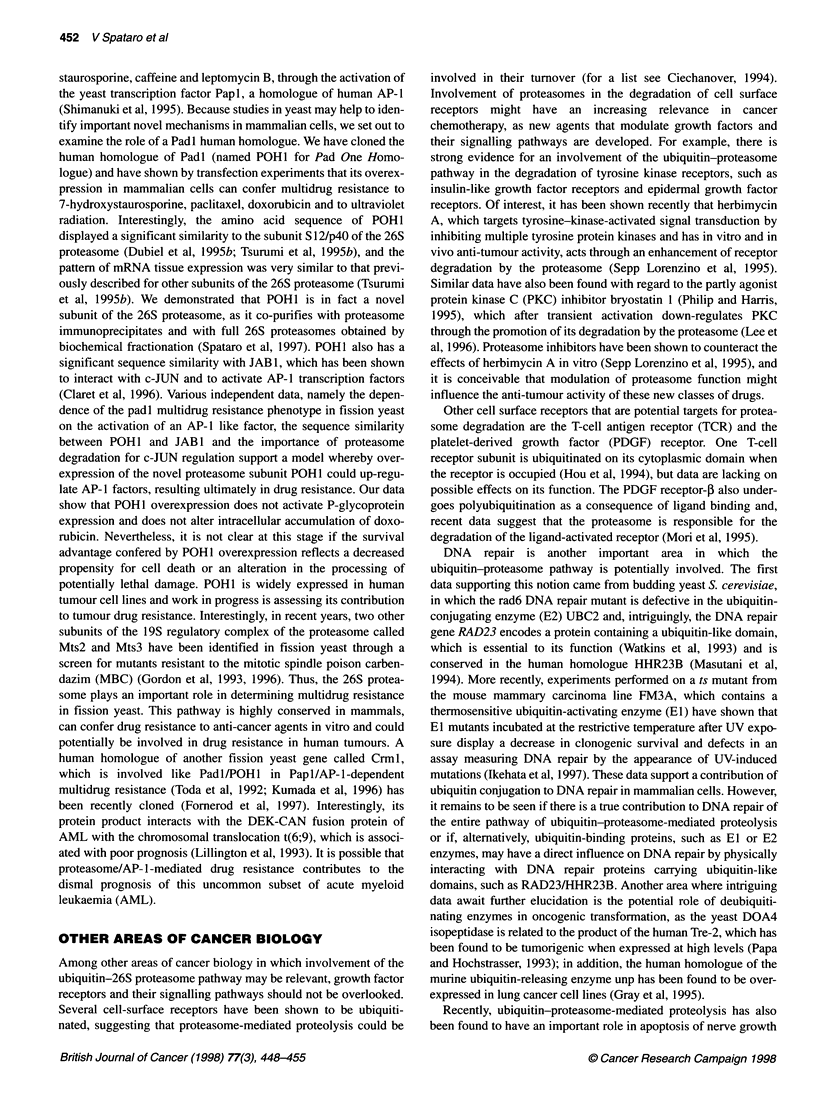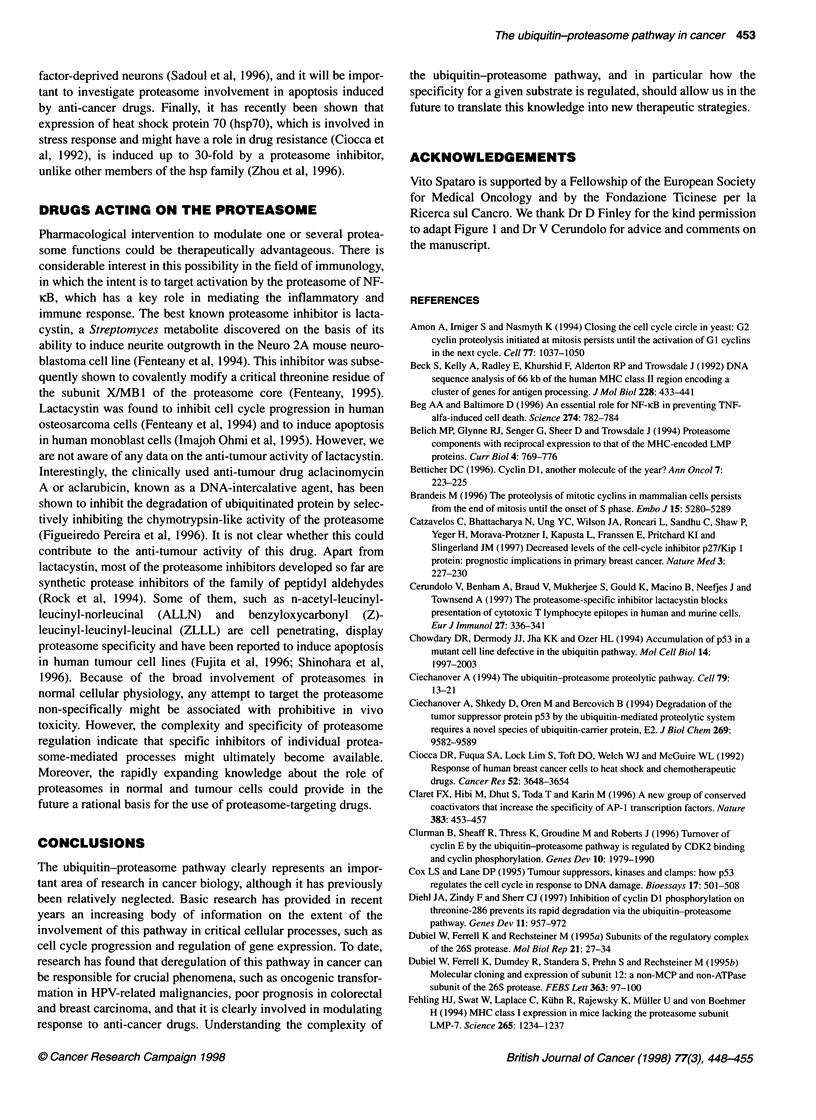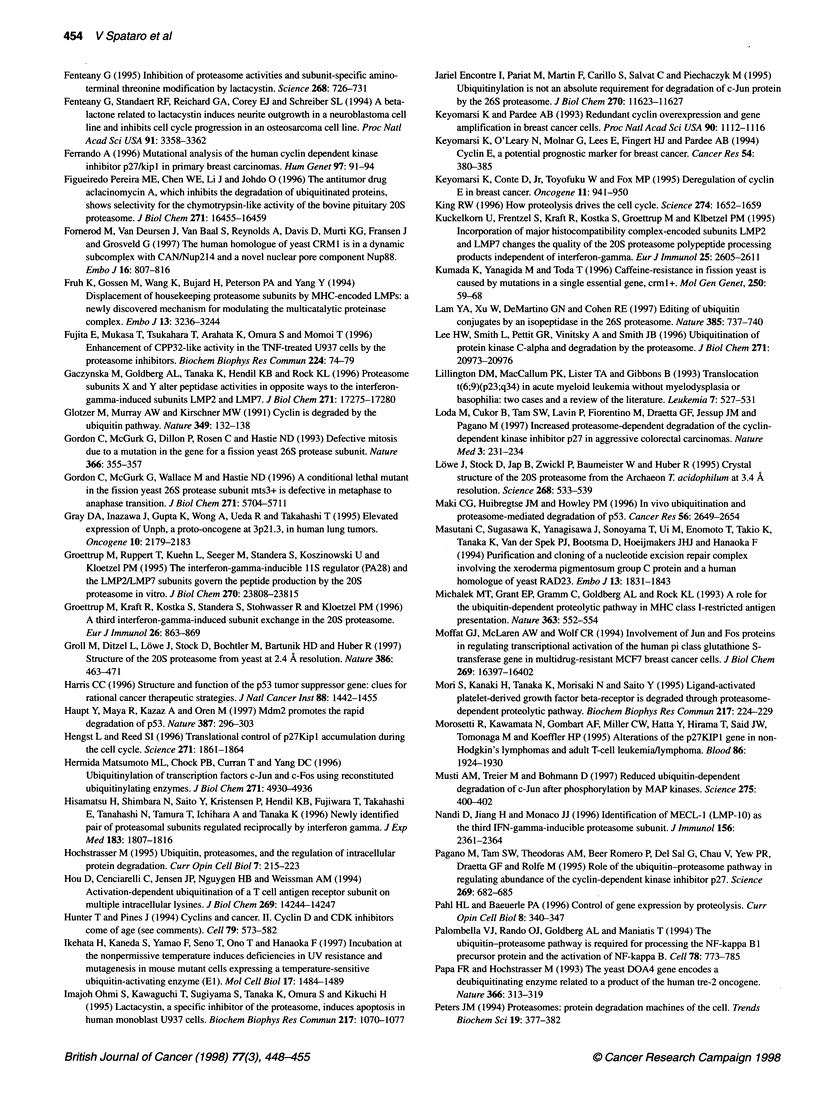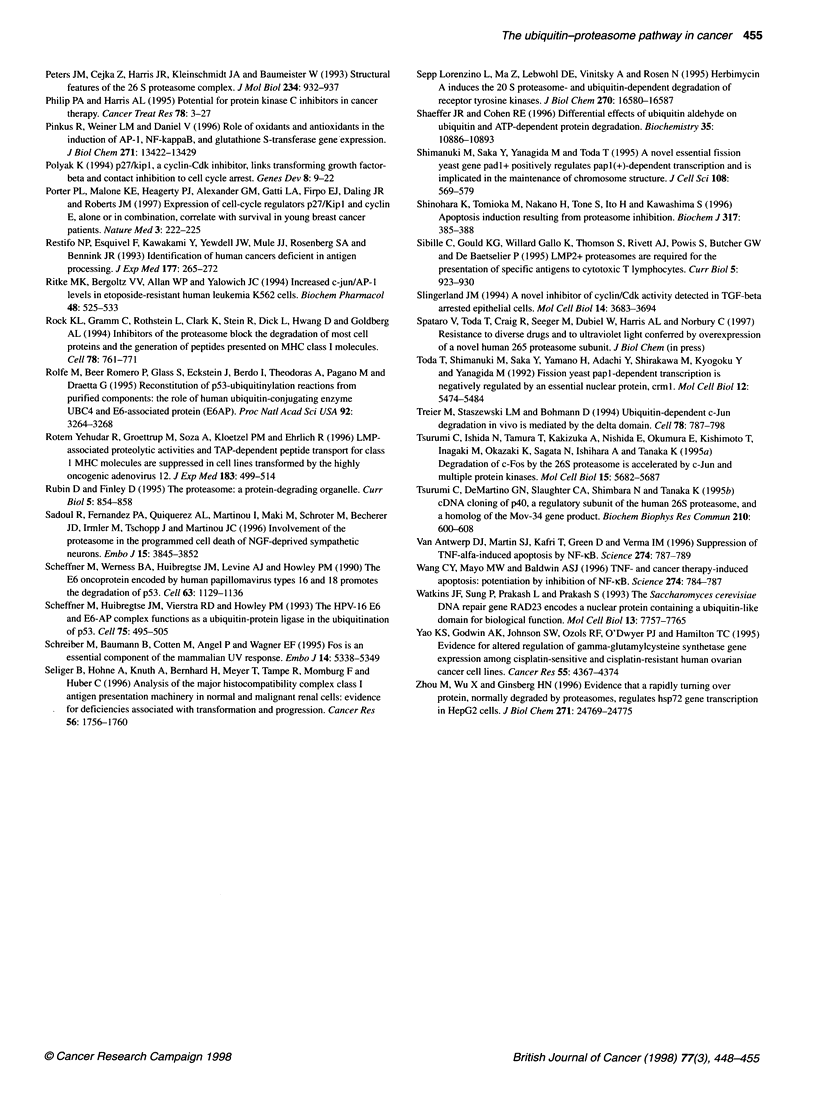# The ubiquitin-proteasome pathway in cancer.

**DOI:** 10.1038/bjc.1998.71

**Published:** 1998

**Authors:** V. Spataro, C. Norbury, A. L. Harris

**Affiliations:** Imperial Cancer Research Fund, University of Oxford, Institute of Molecular Medicine, The John Radcliffe, UK.

## Abstract

Degradation by the 26S proteasome of specific proteins that have been targeted by the ubiquitin pathway is the major intracellular non-lysosomal proteolytic mechanism and is involved in a broad range of processes, such as cell cycle progression, antigen presentation and control of gene expression. Recent work, reviewed here, has shown that this pathway is often the target of cancer-related deregulation and can underlie processes, such as oncogenic transformation, tumour progression, escape from immune surveillance and drug resistance.


British Journal of Cancer (1998) 77(3), 448–455
© 1998 Cancer Research Campaign

### The ubiquitin–proteasome pathway in cancer

**V Spataro, C Norbury and AL Harris**

Imperial Cancer Research Fund, Molecular Oncology Laboratory, University of Oxford, Institute of Molecular Medicine, The John Radcliffe, Headley Way, Oxford OX3 9DS, UK

**Summary** Degradation by the 26S proteasome of specific proteins that have been targeted by the ubiquitin pathway is the major intracellular non-lysosomal proteolytic mechanism and is involved in a broad range of processes, such as cell cycle progression, antigen presentation and control of gene expression. Recent work, reviewed here, has shown that this pathway is often the target of cancer-related deregulation and can underlie processes, such as oncogenic transformation, tumour progression, escape from immune surveillance and drug resistance.

**Keywords:** ubiquitin; proteasome; oncogenesis; drug resistance; immune escape

Eukaryotic cells contain two major proteolytic pathways, namely the lysosomal pathway, which mainly degrades extracellular proteins that have entered the cell via endocytosis or pinocytosis, and the non-lysosomal pathway, which degrades in a cellular particle called the proteasome intracellular proteins, which have been targeted for destruction by a protein called ubiquitin. The ubiquitin–proteasome pathway was initially regarded as a simple mechanism of destruction for old or damaged proteins, but it is now emerging as a crucial mechanism in cellular regulation. Indeed, in recent years it has been found that protein degradation accounts for the regulation of proteins, such as cyclins, cyclin-dependent kinase inhibitors, p53, c-JUN and c-FOS, and it has become increasingly clear that proteolysis is a mechanism of regulation of many cellular processes, including cell cycle progression, transcriptional regulation and antigen presentation (Hochstrasser, 1995; King, 1996; Pahl and Baeuerle, 1996). The importance of proteolysis probably stems from the advantages that it offers over other regulation mechanisms, such as the rapidity of the reduction of the cellular level of a specific protein and the irreversibility of the loss of function after degradation. In addition, it has to be stressed that, by degrading inhibitors or activators of the various pathways, protein degradation can act both as an up-regulation or a down-regulation mechanism.

The ubiquitin–proteasome pathway degrades cytosolic and nuclear proteins via an ATP- and ubiquitin-dependent mechanism, which is centred on a multicatalytic proteinase complex called the 26S proteasome. Substrate proteins are targeted for degradation by the addition of multiple monomers of ubiquitin, a 76 amino acid polypeptide, to specific residues in a multi-step reaction requiring three classes of enzymes called E1, E2 and E3. Initially, a ubiquitin-activating enzyme (E1) activates a ubiquitin monomer at its C-terminal glycine residue to a high-energy thiol ester intermediate. Then, E2 enzymes, also known as ubiquitin-conjugating enzymes (UBC), transfer ubiquitin from E1 to the substrate that is bound to a ubiquitin-protein ligase (E3). The first ubiquitin molecule is usually bound to the substrate by an isopeptide bond between the C-terminal glycine of ubiquitin and an $\epsilon$-$NH_2$ group of a lysine residue of the substrate. The polyubiquitin chain is formed in multiple cycles of this reaction by addition of another ubiquitin molecule to the lysine at position 48 of the previously already conjugated ubiquitin. Release of ubiquitin from the isopeptide linkage with the lysine residue is performed by isopeptidases called ubiquitin C-terminal hydrolases (UCH). Their function is probably important not only in recycling ubiquitin monomers after substrate degradation but also in the recovery of poorly or incorrectly ubiquitinated proteins (Shaeffer and Cohen, 1996).

Polyubiquitinated proteins are substrates for the 26S proteasome. This consists of three large multi-subunit complexes, namely a 700-kDa 20S proteasome core particle and two 19S cap structures, also called PA 700 (for proteasome activator of 700 kDa) (reviewed in Peters, 1994). The 20S particle has the structure of a hollow cylinder composed of four rings of seven related subunits and containing a central channel with three cavities (Löwe et al, 1995; Groll et al 1997). The inner rings are formed of β-subunits, which carry the proteolytically active sites on the inner surface. The outer rings contain subunits that lack proteolytic activity and are thought to control the access to the central cavity. The isolated 20S particle has very limited activity in vitro compared with the 26S proteasome, which is formed by the 20S proteasome with the addition of two 19S/PA700 substructures in opposite orientations, one at each end (Peters et al, 1993), as revealed by electron microscopy (Figure 1). The 19S regulatory complex consists of at least 15 subunits, which can be classified into ATPases and non-ATPases (Dubiel et al, 1995*a*), and is thought to act in recognition, unfolding and translocation of the substrates into the 20S proteasome for proteolysis (Rubin and Finley, 1995). The composition and the function of the regulatory complex is not yet fully characterized and recent data have shown, for example, that the regulatory complex also contains an isopeptidase capable of deubiquitinating substrates (Lam et al, 1997).

Because of the broad involvement of ubiquitin–proteasome proteolysis in fundamental biochemical processes, this pathway is a potential target for cancer-related deregulation, and alterations of proteasome function have indeed been described in events, such as cellular transformation by oncogenic viruses (Scheffner et al,

*Received 20 March 1997*
*Revised 1 July 1997*
*Accepted 8 July 1997*

*Correspondence to:* V Spataro, Servizio Oncologico Ticinese, Ospedale Civico, CH-6900 Lugano, Switzerland

**Figure 1** (Adapted with permission from Rubin and Finley, 1995). The 26S proteasome is a multiprotein complex that acts as a multicatalytic protease degrading proteins that have been targeted by the ubiquitin pathway. Proteins are ubiquitinated in a cascade reaction involving three classes of ubiquitinating enzymes called E1, E2 and E3 and can be deubiquitinated by isopeptidases. The 20S proteasome consists of a stack of four rings of seven subunits. The inner rings made of β-subunits display the catalytic sites on the inner surface. At each end, the 20S proteasome can be capped by a regulatory complex called 19S or PA700, which contains ATPases and is probably involved in recognition, unfolding and translocation of the substrate into the 20S proteasome (Rubin and Finley, 1995)

1990, 1993; Ciechanover et al, 1994) and immune escape (Restifo et al, 1993; Sibille et al, 1995; Rotem Yehudar et al, 1996; Seliger et al, 1996). Furthermore, alterations of proteasome activity in tumour samples have been reported recently to confer in colon and possibly breast cancer a phenotype of clinical aggressiveness associated with poor prognosis (Catzavelos et al, 1997; Loda et al, 1997; Porter et al, 1997). Finally, mutations of proteasome subunits have been found to result in a multidrug resistance phenotype in fission yeast (Gordon et al, 1993, 1996), and we have recently shown that this pathway of multidrug resistance is conserved in mammalian cells (Spataro et al, 1997). Here, we therefore review the rapidly increasing body of information on the role of proteolysis by the ubiquitin/proteasome pathway in various fields of cancer biology.

### p53 AND HPV-RELATED MALIGNANCIES

The product of the tumour-suppressor gene *p53* is an unstable nuclear protein with a half-life of 20–35 min in normal cells. After cellular stress or DNA damage, p53 is stabilized, leading to growth arrest or apoptosis. The rise in p53 protein level is detectable almost immediately after DNA damage, and the absolute level of p53 protein and the duration of the response depend on the nature of the damage (reviewed in Cox and Lane, 1995). This accumulation of p53 is thought to occur mainly via the down-regulation of its degradation by the ubiquitin–proteasome pathway (Harris, 1996; Maki et al, 1996). Although, at present, this supposition has not been experimentally confirmed, it is supported by experimental data in a cell line containing a thermolabile E1 ubiquitin-activating enzyme, in which p53 accumulates at the non-permissive temperature; this accumulation is prevented by introduction of the wild-type *E1* gene (Chowdary et al, 1994). Interestingly, recent evidence suggests that p53 degradation is stimulated by the product of the p53-activated *MDM2* gene (Haupt et al, 1997), providing the basis for a mechanism by which the activation of p53 could be self-limiting. Further research on regulation of p53 by proteolysis is clearly warranted because alterations in this pathway can be functionally equivalent to p53 inactivation. This is well exemplified in the case of human papilloma virus (HPV)-related cancers. The oncogenicity of the human papilloma virus, which is involved in the aetiology of the majority of human anogenital carcinomas, is mediated by up-regulation of p53 degradation by the ubiquitin–proteasome pathway. The E6 oncoprotein encoded by high-risk HPV (e.g. HPV-16, -18, -5 and -8) binds to p53 and promotes its degradation by the proteasome (Scheffner et al, 1990) – a property that is critical for immortalization of human cells by HPV. In contrast, low-risk HPV (e.g. HPV-6 and -11) encode an E6 protein that does not bind to p53 and does not promote its degradation. The formation of the E6–p53 complex requires a cellular E6-binding protein called E6-AP (E6-associated protein) (Scheffner et al, 1993), which forms thiol ester complexes with ubiquitin in the presence of enzymes of the E2 category, such as UBC4 (Rolfe et al, 1995) or E2-F1 (Ciechanover et al, 1994). E6-AP acts as an E3 enzyme, which ubiquitinates p53, leading to its rapid degradation by the 26S proteasome.

### p27 AS A PROGNOSTIC FACTOR

Progression through the cell cycle is promoted by oscillation in the activity of cyclin-dependent kinases (CDK), and proteolysis by the ubiquitin–proteasome pathway regulates CDK activity by degrading CDK activators and inhibitors. Furthermore, proteolysis by the proteasome is crucial during mitosis in triggering the transition from metaphase to anaphase (reviewed in King, 1996). Among the substrates for proteolysis in the cell cycle machinery, clinically important data are emerging with regard to the CDK inhibitor p27. p27 inhibits a wide variety of cyclin–CDK complexes in vitro and its activity is up-regulated by cytokines, such as TGF-β and by cell–cell contact, linking extracellular signals to the cell cycle (Polyak, 1994; Slingerland, 1994). Loss of contact inhibition and of response to TGF-β in transformed cells may imply an alteration of function of p27 during oncogenesis, even though p27 mutations in human tumours are extremely rare (Hunter and Pines, 1994; Morosetti et al, 1995; Ferrando, 1996). Unlike p21, which is also a member of the family of cip/kip CDK inhibitors acting in $G_1$ and appears to be regulated principally at the transcriptional level, p27 is critically regulated post-translationally by proteolysis by the ubiquitin–proteasome pathway (Pagano et al, 1995; Hengst and Reed, 1996). Recently, it has been found that low p27 protein levels in common tumours, such as colorectal carcinomas and breast cancer, are associated with a poor prognosis (Catzavelos et al, 1997; Loda et al, 1997; Porter et al, 1997). In both tumour types (Catzavelos et al, 1997; Loda et al, 1997), comparison of immunohistochemical analysis and in situ hybridization showed a discordance between p27 mRNA and protein levels, suggesting that, also in tumours, p27 levels could be regulated post-translationally. Moreover, in one of the studies, it was clearly shown that increased proteasome-dependent degradation was responsible for low p27

© Cancer Research Campaign 1998

Table 1 Summary of settings in which the ubiquitin–proteasome pathway plays a role in cancer (see text for references)

| Substrate | Proteolysis dysregulation | Functional effect | Biological effect | Comment |
|---|---|---|---|---|
| p53 | Increased | p53 Inactivation | Transformation in HPV-related malignancies | Mediated by E6 oncoprotein of 'high-risk' HPV |
| p27 | Increased | p27 Inactivation | Tumour progression | Unfavourable prognosis in retrospective studies in colon and breast cancer |
| Cyclin D1, E and B | Decreased? | Cyclin D1, E or B overexpression | Tumour progression | Overexpression in tumour cell lines and surgical specimens, contribution of decreased degradation? |
| MHC-I-restricted | Decreased | Defective antigen presentation | Escape from immune surveillance | Suggested by in vitro studies, preliminary in vivo evidence |
| NF-κB inhibitor, IκB | Increased | Increased NF-κB activation | Resistance to TNF-α killing | Overcome by proteasome inhibitors |
| Transcription factors of the AP-1 family | Decreased | Increased AP-1 activity | Multidrug resistance | Evidence in yeast, highly conserved pathway in human, role in tumour drug resistance? |

levels in tumour samples of colorectal carcinomas. Total cellular extracts from frozen tumour samples were tested for p27 proteasome-mediated degradation using recombinant p27 as a substrate, and a very good correlation was found between low levels of p27 and increased proteasome activity. Degradation was abolished by proteasome depletion and resumed after proteasome readdition (Loda et al, 1997). Down-regulation of p27 by the proteasome was found in tumours regardless of clinical stage. In breast cancer, Catzavelos et al (1997) showed that increased p27 proteolysis can be an early event in tumorigenesis, as suggested by analysis of high-grade ductal carcinoma in situ (DCIS) or, alternatively, can occur upon progression, as shown by reduced p27 levels in axillary lymph node metastasis compared with primary tumours assessed simultaneously. Even taking into account the caveats associated with retrospective studies on prognostic factors, these three recent studies (Catzavelos et al, 1997; Loda et al, 1997; Porter et al, 1997) conclude that p27 protein level (and its proteasome-dependent degradation, which was shown to be inversely related) are powerful independent prognostic factors of survival in both tumour types and show clearly that deregulation of gene products involved in clinical tumour progression can occur via alterations of ubiquitin–proteasome proteolysis. Furthermore, they show that this is not a rare event, given that the unfavourable phenotype of decreased p27 levels (defined as immunostaining in < 50% of the cells or as a score of staining of 0–1 on a 0 to 6 scale) involves the majority of the studied population for both colorectal cancer and breast cancer (Catzavelos et al, 1997; Loda et al, 1997; Porter et al, 1997). Thus the frequency of the phenotype of decreased p27 and its distribution, which is independent of most other prognostic factors, make p27 levels a very promising new prognostic factor to be evaluated further. Loda et al (1997) have shown that, perhaps unexpectedly, p27 degradation activity is not correlated with degradation by the proteasome of other substrates, such as p21 and cyclin A, which underscores that the substrate specificity of the ubiquitin–proteasome pathway is highly regulated (Hochstrasser, 1995). Identification of the element(s) responsible for targeting p27 to the ubiquitin–proteasome pathway would, of course, be extremely important for unravelling this novel pathway associated with tumour progression.

Like p27, other elements of the cell cycle machinery that are substrates of ubiquitin–proteasome degradation are potential targets for deregulation in tumours. One of the best characterized transitions in the normal cell cycle is the rapid proteasome-mediated degradation of cyclin B at the exit from mitosis (Glotzer et al, 1991), and recent evidence shows that continuing rapid proteolysis accounts for the low levels of cyclin B until the onset of S phase (Amon et al, 1994; Brandeis, 1996). Cyclin B has been found to be overexpressed in a set of breast cancer cell lines (Keyomarsi and Pardee, 1993), and it would be interesting to assess whether or not decreased proteolysis by the proteasome is involved in its overexpression. Similarly, cyclin E has been found to be overexpressed in breast cancer cell lines and in surgical specimens of breast tumours (Keyomarsi et al, 1994, 1995), and cyclin D1 is frequently overexpressed in many common tumour types (Betticher, 1996). Recent evidence suggests that cyclin D1 and E are substrates of the ubiquitin–proteasome pathway (Clurman et al, 1996; Diehl et al, 1997), and decreases in their degradation could contribute to the overexpression of these cyclins in tumours.

### ANTIGEN PRESENTATION

The 26S proteasome is responsible for the processing of MHC-restricted class I antigens. Peptides derived from endogenously expressed cytoplasmic proteins are carried by MHC class I molecules from the endoplasmic reticulum to the surface for recognition by cytotoxic T lymphocytes. The proteasome was postulated to be the proteolytic system that degrades cytosolic proteins, when it was found that the genes encoding subunits LMP-2 and LMP-7 of the

 © Cancer Research Campaign 1998

proteasome complex were included in the MHC gene cluster (see for example Beck et al, 1992). Experiments performed in a mutant cell with a thermolabile E1-ubiquitinating enzyme (Michalek et al, 1993) and with proteasome inhibitors (Rock et al, 1994; Cerundolo et al, 1997) have subsequently demonstrated that the proteasome is necessary for class I-restricted antigen presentation. This is confirmed by the analysis of mice lacking LMP-7, which have decreased surface expression of MHC class I molecules and present antigens inefficiently (Fehling et al, 1994). It has also been shown that 3 of the 28 subunits composing the 20S catalytic core, namely subunits X, Y and Z, are interchangeable with the alternative subunits LMP2, LMP7 and LMP10 respectively (Belich et al, 1994; Fruh et al, 1994; Groettrup et al, 1996; Hisamatsu et al, 1996; Nandi et al, 1996) upon induction by interferon-γ. These substitutions result in an enhancement of peptidase activity, a change in the quality of generated peptides (Gaczynska et al, 1996; Kuckelkorn et al, 1995) and eventually in a more efficient antigen presentation. Interferon-γ also induces the binding to the 20S catalytic core of the proteasome of a complex called 11S regulator or PA28, which may further increase the spectrum of peptides generated (Groettrup et al, 1995). There is strong evidence that MHC class I-restricted peptide presentation is modified in tumours and may contribute to escape from immune surveillance. Alterations of ubiquitin–proteasome degradation have been reported among other alterations in this pathway. Three different small-cell lung carcinoma lines with low to undetectable levels of mRNA for LMP2 and LMP7 and functional deficiencies in antigen presentation have been described (Restifo et al, 1993). The mouse T-cell lymphoma line SP-3 displays underexpression of LMP-2 and is defective for antigen presentation, whereas LMP-2 expression and antigen presentation to cytotoxic T lymphocytes are restored upon expression of interferon-γ by transfection (Sibille et al, 1995). Similar studies on tumour samples are rare. An analysis of expression of both LMP-2 and LMP-7 proteasome subunits together with other elements of the antigen presentation machinery has been carried out on a primary renal cancer and a lymph node metastasis of the same patient and compared with normal kidney. Deficiencies at all levels, including the expression of LMP-2 and LMP-7 proteasome subunits, were associated with transformation and progression. Interferon-α and, in particular, interferon-γ could partly suppress these defects (Seliger et al, 1996). The potential importance of subunits LMP-2 and LMP-7 for MHC class I-restricted antigen presentation is also underscored by the fact that they are specifically down-regulated after viral transformation in vitro by oncogenic viruses (Rotem Yehudar et al, 1996).

### REGULATION OF TRANSCRIPTION FACTORS BY PROTEOLYSIS

Increasing evidence shows that the proteasome also participates in events that control gene transcription. Several transcriptional regulators, including nuclear factor-kappa B (NF-κB), p53 (see above), c-JUN, sterol-regulated element-binding proteins and MATα2 have been recently shown to be regulated by proteolysis, either for the activation or the inactivation of gene expression (for a review see Pahl and Baeuerle, 1996).

NF-κB is involved in the activation of genes encoding products such as cytokines, chemokines, growth factors, cell-adhesion molecules and surface receptors in response to a great variety of pathogenic signals and therefore has a central role in mediating the immune/inflammatory responses. NF-κB has been reported to be activated by the cytotoxic agents TNF-α, daunorubicin, etoposide, ionizing radiation or oxidative stress but not by the protein kinase C inhibitor staurosporine (Wang et al, 1996). The activation of NF-κB requires two steps of proteasome-dependent proteolysis. Active NF-κB is a nuclear heterodimer consisting of two subunits called p50 and p65. Ubiquitin–proteasome proteolysis is involved first in the biogenesis of the subunit p50 from the precursor p105 and then in the cytoplasmic degradation of the inhibitory factor IκB, which allows the translocation of the active dimer into the nucleus (Palombella et al, 1994). Recently published data attribute an anti-apoptotic role to NF-κB in response to some cytotoxic agents (Beg and Baltimore, 1996; Van Antwerp et al, 1996; Wang et al, 1996). In one case, TNF-α was more toxic for immortalized embryonic cells of NF-κB knock-out mice than for controls (Beg and Baltimore, 1996) and in other experiments expression of the super-repressor IκB-a (inhibiting NF-κB activation) moderately increased the sensitivity to TNF-α, daunorubicin and ionizing radiation (Wang et al, 1996). Consistent with this, the proteasome inhibitor MG132 (preventing NF-κB activation) strongly enhanced, in a dose-dependent fashion, the killing of HT1080V cells by TNF-α. The proto-oncogene products c-JUN and c-FOS constitute the transcription factor AP-1 (for activator protein 1) either as heterodimers or as c-JUN homodimers and are well-known substrates for ubiquitin–proteasome degradation (Treier et al, 1994; Jariel Encontre et al, 1995; Tsurumi et al, 1995*a*; Hermida Matsumoto et al, 1996; Musti et al, 1997). The degradation of c-JUN is dependent on a segment of 27 amino acids called the delta domain, which is necessary for both ubiquitination and degradation. The delta region, and hence this mechanism of down-regulation, is lost in v-JUN, the transforming retroviral counterpart of c-JUN, and this increased stability very likely contributes to its oncogenicity (Treier et al, 1994). Moreover, it has been convincingly shown that c-JUN is degraded by this pathway, but recent data suggest that ubiquitin–proteasome-mediated proteolysis of c-JUN could play an essential role in regulation of activity of AP-1 factors (Musti et al, 1997). There is a high degree of regulation of c-JUN proteolysis, with the presence of c-FOS and dimerization itself influencing the ubiquitination and the degradation activity (Tsurumi et al, 1995*a*; Hermida Matsumoto et al, 1996). Like NF-κB, AP-1 factors are important in the cellular response to oxidative stress (Schreiber et al, 1995; Pinkus et al, 1996) and are involved in the induction of a variety of genes encoding important enzymes in glutathione-related detoxification pathways, such as the isozymes α, π and γ of glutathione-*S*-transferase and γ-glutamyl-cysteine synthetase. Up-regulation of AP-1 activity has been associated with drug resistance in several instances, such as in a multidrug-resistant derivative of MCF7 cells obtained after vincristine selection (Moffat et al, 1994) in etoposide-resistant human leukaemia cell lines (Ritke et al, 1994) and in cisplatin-resistant ovarian cancer lines (Yao et al, 1995). Given the relevance of proteolysis for c-JUN regulation, this acquires particular importance in the light of recent data discussed in the following section that link the proteasome, AP-1 factors and multidrug resistance (Spataro et al, 1997).

### DRUG RESISTANCE

We recently identified a novel component of the 26S proteasome that indicates a link between ubiquitin-dependent proteolysis and drug resistance. Overexpression of the fission yeast Pad1 protein confers multidrug resistance to unrelated compounds, such as

© Cancer Research Campaign 1998 

staurosporine, caffeine and leptomycin B, through the activation of the yeast transcription factor Pap1, a homologue of human AP-1 (Shimanuki et al, 1995). Because studies in yeast may help to identify important novel mechanisms in mammalian cells, we set out to examine the role of a Pad1 human homologue. We have cloned the human homologue of Pad1 (named POH1 for *P*ad *O*ne *H*omologue) and have shown by transfection experiments that its overexpression in mammalian cells can confer multidrug resistance to 7-hydroxystaurosporine, paclitaxel, doxorubicin and to ultraviolet radiation. Interestingly, the amino acid sequence of POH1 displayed a significant similarity to the subunit S12/p40 of the 26S proteasome (Dubiel et al, 1995*b*; Tsurumi et al, 1995*b*), and the pattern of mRNA tissue expression was very similar to that previously described for other subunits of the 26S proteasome (Tsurumi et al, 1995*b*). We demonstrated that POH1 is in fact a novel subunit of the 26S proteasome, as it co-purifies with proteasome immunoprecipitates and with full 26S proteasomes obtained by biochemical fractionation (Spataro et al, 1997). POH1 also has a significant sequence similarity with JAB1, which has been shown to interact with c-JUN and to activate AP-1 transcription factors (Claret et al, 1996). Various independent data, namely the dependence of the pad1 multidrug resistance phenotype in fission yeast on the activation of an AP-1 like factor, the sequence similarity between POH1 and JAB1 and the importance of proteasome degradation for c-JUN regulation support a model whereby overexpression of the novel proteasome subunit POH1 could up-regulate AP-1 factors, resulting ultimately in drug resistance. Our data show that POH1 overexpression does not activate P-glycoprotein expression and does not alter intracellular accumulation of doxorubicin. Nevertheless, it is not clear at this stage if the survival advantage confered by POH1 overexpression reflects a decreased propensity for cell death or an alteration in the processing of potentially lethal damage. POH1 is widely expressed in human tumour cell lines and work in progress is assessing its contribution to tumour drug resistance. Interestingly, in recent years, two other subunits of the 19S regulatory complex of the proteasome called Mts2 and Mts3 have been identified in fission yeast through a screen for mutants resistant to the mitotic spindle poison carbendazim (MBC) (Gordon et al, 1993, 1996). Thus, the 26S proteasome plays an important role in determining multidrug resistance in fission yeast. This pathway is highly conserved in mammals, can confer drug resistance to anti-cancer agents in vitro and could potentially be involved in drug resistance in human tumours. A human homologue of another fission yeast gene called Crm1, which is involved like Pad1/POH1 in Pap1/AP-1-dependent multidrug resistance (Toda et al, 1992; Kumada et al, 1996) has been recently cloned (Fornerod et al, 1997). Interestingly, its protein product interacts with the DEK-CAN fusion protein of AML with the chromosomal translocation t(6;9), which is associated with poor prognosis (Lillington et al, 1993). It is possible that proteasome/AP-1-mediated drug resistance contributes to the dismal prognosis of this uncommon subset of acute myeloid leukaemia (AML).

### OTHER AREAS OF CANCER BIOLOGY

Among other areas of cancer biology in which involvement of the ubiquitin–26S proteasome pathway may be relevant, growth factor receptors and their signalling pathways should not be overlooked. Several cell-surface receptors have been shown to be ubiquitinated, suggesting that proteasome-mediated proteolysis could be involved in their turnover (for a list see Ciechanover, 1994). Involvement of proteasomes in the degradation of cell surface receptors might have an increasing relevance in cancer chemotherapy, as new agents that modulate growth factors and their signalling pathways are developed. For example, there is strong evidence for an involvement of the ubiquitin–proteasome pathway in the degradation of tyrosine kinase receptors, such as insulin-like growth factor receptors and epidermal growth factor receptors. Of interest, it has been shown recently that herbimycin A, which targets tyrosine–kinase-activated signal transduction by inhibiting multiple tyrosine protein kinases and has in vitro and in vivo anti-tumour activity, acts through an enhancement of receptor degradation by the proteasome (Sepp Lorenzino et al, 1995). Similar data have also been found with regard to the partly agonist protein kinase C (PKC) inhibitor bryostatin 1 (Philip and Harris, 1995), which after transient activation down-regulates PKC through the promotion of its degradation by the proteasome (Lee et al, 1996). Proteasome inhibitors have been shown to counteract the effects of herbimycin A in vitro (Sepp Lorenzino et al, 1995), and it is conceivable that modulation of proteasome function might influence the anti-tumour activity of these new classes of drugs.

Other cell surface receptors that are potential targets for proteasome degradation are the T-cell antigen receptor (TCR) and the platelet-derived growth factor (PDGF) receptor. One T-cell receptor subunit is ubiquitinated on its cytoplasmic domain when the receptor is occupied (Hou et al, 1994), but data are lacking on possible effects on its function. The PDGF receptor-β also undergoes polyubiquitination as a consequence of ligand binding and, recent data suggest that the proteasome is responsible for the degradation of the ligand-activated receptor (Mori et al, 1995).

DNA repair is another important area in which the ubiquitin–proteasome pathway is potentially involved. The first data supporting this notion came from budding yeast *S. cerevisiae*, in which the rad6 DNA repair mutant is defective in the ubiquitin-conjugating enzyme (E2) UBC2 and, intriguingly, the DNA repair gene *RAD23* encodes a protein containing a ubiquitin-like domain, which is essential to its function (Watkins et al, 1993) and is conserved in the human homologue HHR23B (Masutani et al, 1994). More recently, experiments performed on a *ts* mutant from the mouse mammary carcinoma line FM3A, which contains a thermosensitive ubiquitin-activating enzyme (E1) have shown that E1 mutants incubated at the restrictive temperature after UV exposure display a decrease in clonogenic survival and defects in an assay measuring DNA repair by the appearance of UV-induced mutations (Ikehata et al, 1997). These data support a contribution of ubiquitin conjugation to DNA repair in mammalian cells. However, it remains to be seen if there is a true contribution to DNA repair of the entire pathway of ubiquitin–proteasome-mediated proteolysis or if, alternatively, ubiquitin-binding proteins, such as E1 or E2 enzymes, may have a direct influence on DNA repair by physically interacting with DNA repair proteins carrying ubiquitin-like domains, such as RAD23/HHR23B. Another area where intriguing data await further elucidation is the potential role of deubiquitinating enzymes in oncogenic transformation, as the yeast DOA4 isopeptidase is related to the product of the human Tre-2, which has been found to be tumorigenic when expressed at high levels (Papa and Hochstrasser, 1993); in addition, the human homologue of the murine ubiquitin-releasing enzyme unp has been found to be overexpressed in lung cancer cell lines (Gray et al, 1995).

Recently, ubiquitin–proteasome-mediated proteolysis has also been found to have an important role in apoptosis of nerve growth

© Cancer Research Campaign 1998

factor-deprived neurons (Sadoul et al, 1996), and it will be important to investigate proteasome involvement in apoptosis induced by anti-cancer drugs. Finally, it has recently been shown that expression of heat shock protein 70 (hsp70), which is involved in stress response and might have a role in drug resistance (Ciocca et al, 1992), is induced up to 30-fold by a proteasome inhibitor, unlike other members of the hsp family (Zhou et al, 1996).

### DRUGS ACTING ON THE PROTEASOME

Pharmacological intervention to modulate one or several proteasome functions could be therapeutically advantageous. There is considerable interest in this possibility in the field of immunology, in which the intent is to target activation by the proteasome of NF-κB, which has a key role in mediating the inflammatory and immune response. The best known proteasome inhibitor is lactacystin, a *Streptomyces* metabolite discovered on the basis of its ability to induce neurite outgrowth in the Neuro 2A mouse neuroblastoma cell line (Fenteany et al, 1994). This inhibitor was subsequently shown to covalently modify a critical threonine residue of the subunit X/MB1 of the proteasome core (Fenteany, 1995). Lactacystin was found to inhibit cell cycle progression in human osteosarcoma cells (Fenteany et al, 1994) and to induce apoptosis in human monoblast cells (Imajoh Ohmi et al, 1995). However, we are not aware of any data on the anti-tumour activity of lactacystin. Interestingly, the clinically used anti-tumour drug aclacinomycin A or aclarubicin, known as a DNA-intercalative agent, has been shown to inhibit the degradation of ubiquitinated protein by selectively inhibiting the chymotrypsin-like activity of the proteasome (Figueiredo Pereira et al, 1996). It is not clear whether this could contribute to the anti-tumour activity of this drug. Apart from lactacystin, most of the proteasome inhibitors developed so far are synthetic protease inhibitors of the family of peptidyl aldehydes (Rock et al, 1994). Some of them, such as n-acetyl-leucinyl-leucinyl-norleucinal (ALLN) and benzyloxycarbonyl (Z)-leucinyl-leucinyl-leucinal (ZLLL) are cell penetrating, display proteasome specificity and have been reported to induce apoptosis in human tumour cell lines (Fujita et al, 1996; Shinohara et al, 1996). Because of the broad involvement of proteasomes in normal cellular physiology, any attempt to target the proteasome non-specifically might be associated with prohibitive in vivo toxicity. However, the complexity and specificity of proteasome regulation indicate that specific inhibitors of individual proteasome-mediated processes might ultimately become available. Moreover, the rapidly expanding knowledge about the role of proteasomes in normal and tumour cells could provide in the future a rational basis for the use of proteasome-targeting drugs.

### CONCLUSIONS

The ubiquitin–proteasome pathway clearly represents an important area of research in cancer biology, although it has previously been relatively neglected. Basic research has provided in recent years an increasing body of information on the extent of the involvement of this pathway in critical cellular processes, such as cell cycle progression and regulation of gene expression. To date, research has found that deregulation of this pathway in cancer can be responsible for crucial phenomena, such as oncogenic transformation in HPV-related malignancies, poor prognosis in colorectal and breast carcinoma, and that it is clearly involved in modulating response to anti-cancer drugs. Understanding the complexity of the ubiquitin–proteasome pathway, and in particular how the specificity for a given substrate is regulated, should allow us in the future to translate this knowledge into new therapeutic strategies.

### ACKNOWLEDGEMENTS

Vito Spataro is supported by a Fellowship of the European Society for Medical Oncology and by the Fondazione Ticinese per la Ricerca sul Cancro. We thank Dr D Finley for the kind permission to adapt Figure 1 and Dr V Cerundolo for advice and comments on the manuscript.

### REFERENCES

Amon A, Irniger S and Nasmyth K (1994) Closing the cell cycle circle in yeast: G2 cyclin proteolysis initiated at mitosis persists until the activation of G1 cyclins in the next cycle. *Cell* **77**: 1037–1050

Beck S, Kelly A, Radley E, Khurshid F, Alderton RP and Trowsdale J (1992) DNA sequence analysis of 66 kb of the human MHC class II region encoding a cluster of genes for antigen processing. *J Mol Biol* **228**: 433–441

Beg AA and Baltimore D (1996) An essential role for NF-κB in preventing TNF-alfa-induced cell death. *Science* **274**: 782–784

Belich MP, Glynne RJ, Senger G, Sheer D and Trowsdale J (1994) Proteasome components with reciprocal expression to that of the MHC-encoded LMP proteins. *Curr Biol* **4**: 769–776

Betticher DC (1996). Cyclin D1, another molecule of the year? *Ann Oncol* **7**: 223–225

Brandeis M (1996) The proteolysis of mitotic cyclins in mammalian cells persists from the end of mitosis until the onset of S phase. *Embo J* **15**: 5280–5289

Catzavelos C, Bhattacharya N, Ung YC, Wilson JA, Roncari L, Sandhu C, Shaw P, Yeger H, Morava-Protzner I, Kapusta L, Franssen E, Pritchard KI and Slingerland JM (1997) Decreased levels of the cell-cycle inhibitor p27/Kip 1 protein: prognostic implications in primary breast cancer. *Nature Med* **3**: 227–230

Cerundolo V, Benham A, Braud V, Mukherjee S, Gould K, Macino B, Neefjes J and Townsend A (1997) The proteasome-specific inhibitor lactacystin blocks presentation of cytotoxic T lymphocyte epitopes in human and murine cells. *Eur J Immunol* **27**: 336–341

Chowdary DR, Dermody JJ, Jha KK and Ozer HL (1994) Accumulation of p53 in a mutant cell line defective in the ubiquitin pathway. *Mol Cell Biol* **14**: 1997–2003

Ciechanover A (1994) The ubiquitin–proteasome proteolytic pathway. *Cell* **79**: 13–21

Ciechanover A, Shkedy D, Oren M and Bercovich B (1994) Degradation of the tumor suppressor protein p53 by the ubiquitin-mediated proteolytic system requires a novel species of ubiquitin-carrier protein, E2. *J Biol Chem* **269**: 9582–9589

Ciocca DR, Fuqua SA, Lock Lim S, Toft DO, Welch WJ and McGuire WL (1992) Response of human breast cancer cells to heat shock and chemotherapeutic drugs. *Cancer Res* **52**: 3648–3654

Claret FX, Hibi M, Dhut S, Toda T and Karin M (1996) A new group of conserved coactivators that increase the specificity of AP-1 transcription factors. *Nature* **383**: 453–457

Clurman B, Sheaff R, Thress K, Groudine M and Roberts J (1996) Turnover of cyclin E by the ubiquitin–proteasome pathway is regulated by CDK2 binding and cyclin phosphorylation. *Genes Dev* **10**: 1979–1990

Cox LS and Lane DP (1995) Tumour suppressors, kinases and clamps: how p53 regulates the cell cycle in response to DNA damage. *Bioessays* **17**: 501–508

Diehl JA, Zindy F and Sherr CJ (1997) Inhibition of cyclin D1 phosphorylation on threonine-286 prevents its rapid degradation via the ubiquitin–proteasome pathway. *Genes Dev* **11**: 957–972

Dubiel W, Ferrell K and Rechsteiner M (1995*a*) Subunits of the regulatory complex of the 26S protease. *Mol Biol Rep* **21**: 27–34

Dubiel W, Ferrell K, Dumdey R, Standera S, Prehn S and Rechsteiner M (1995*b*) Molecular cloning and expression of subunit 12: a non-MCP and non-ATPase subunit of the 26S protease. *FEBS Lett* **363**: 97–100

Fehling HJ, Swat W, Laplace C, Kühn R, Rajewsky K, Müller U and von Boehmer H (1994) MHC class I expression in mice lacking the proteasome subunit LMP-7. *Science* **265**: 1234–1237

© Cancer Research Campaign 1998 

Fenteany G (1995) Inhibition of proteasome activities and subunit-specific amino-terminal threonine modification by lactacystin. *Science* **268**: 726–731

Fenteany G, Standaert RF, Reichard GA, Corey EJ and Schreiber SL (1994) A beta-lactone related to lactacystin induces neurite outgrowth in a neuroblastoma cell line and inhibits cell cycle progression in an osteosarcoma cell line. *Proc Natl Acad Sci USA* **91**: 3358–3362

Ferrando A (1996) Mutational analysis of the human cyclin dependent kinase inhibitor p27/kip1 in primary breast carcinomas. *Hum Genet* **97**: 91–94

Figueiredo Pereira ME, Chen WE, Li J and Johdo O (1996) The antitumor drug aclacinomycin A, which inhibits the degradation of ubiquitinated proteins, shows selectivity for the chymotrypsin-like activity of the bovine pituitary 20S proteasome. *J Biol Chem* **271**: 16455–16459

Fornerod M, Van Deursen J, Van Baal S, Reynolds A, Davis D, Murti KG, Fransen J and Grosveld G (1997) The human homologue of yeast CRM1 is in a dynamic subcomplex with CAN/Nup214 and a novel nuclear pore component Nup88. *Embo J* **16**: 807–816

Fruh K, Gossen M, Wang K, Bujard H, Peterson PA and Yang Y (1994) Displacement of housekeeping proteasome subunits by MHC-encoded LMPs: a newly discovered mechanism for modulating the multicatalytic proteinase complex. *Embo J* **13**: 3236–3244

Fujita E, Mukasa T, Tsukahara T, Arahata K, Omura S and Momoi T (1996) Enhancement of CPP32-like activity in the TNF-treated U937 cells by the proteasome inhibitors. *Biochem Biophys Res Commun* **224**: 74–79

Gaczynska M, Goldberg AL, Tanaka K, Hendil KB and Rock KL (1996) Proteasome subunits X and Y alter peptidase activities in opposite ways to the interferon-gamma-induced subunits LMP2 and LMP7. *J Biol Chem* **271**: 17275–17280

Glotzer M, Murray AW and Kirschner MW (1991) Cyclin is degraded by the ubiquitin pathway. *Nature* **349**: 132–138

Gordon C, McGurk G, Dillon P, Rosen C and Hastie ND (1993) Defective mitosis due to a mutation in the gene for a fission yeast 26S protease subunit. *Nature* **366**: 355–357

Gordon C, McGurk G, Wallace M and Hastie ND (1996) A conditional lethal mutant in the fission yeast 26S protease subunit mts3+ is defective in metaphase to anaphase transition. *J Biol Chem* **271**: 5704–5711

Gray DA, Inazawa J, Gupta K, Wong A, Ueda R and Takahashi T (1995) Elevated expression of Unph, a proto-oncogene at 3p21.3, in human lung tumors. *Oncogene* **10**: 2179–2183

Groettrup M, Ruppert T, Kuehn L, Seeger M, Standera S, Koszinowski U and Kloetzel PM (1995) The interferon-gamma-inducible 11S regulator (PA28) and the LMP2/LMP7 subunits govern the peptide production by the 20S proteasome in vitro. *J Biol Chem* **270**: 23808–23815

Groettrup M, Kraft R, Kostka S, Standera S, Stohwasser R and Kloetzel PM (1996) A third interferon-gamma-induced subunit exchange in the 20S proteasome. *Eur J Immunol* **26**: 863–869

Groll M, Ditzel L, Löwe J, Stock D, Bochtler M, Bartunik HD and Huber R (1997) Structure of the 20S proteasome from yeast at 2.4 Å resolution. *Nature* **386**: 463–471

Harris CC (1996) Structure and function of the p53 tumor suppressor gene: clues for rational cancer therapeutic strategies. *J Natl Cancer Inst* **88**: 1442–1455

Haupt Y, Maya R, Kazaz A and Oren M (1997) Mdm2 promotes the rapid degradation of p53. *Nature* **387**: 296–303

Hengst L and Reed SI (1996) Translational control of p27Kip1 accumulation during the cell cycle. *Science* **271**: 1861–1864

Hermida Matsumoto ML, Chock PB, Curran T and Yang DC (1996) Ubiquitinylation of transcription factors c-Jun and c-Fos using reconstituted ubiquitinylating enzymes. *J Biol Chem* **271**: 4930–4936

Hisamatsu H, Shimbara N, Saito Y, Kristensen P, Hendil KB, Fujiwara T, Takahashi E, Tanahashi N, Tamura T, Ichihara A and Tanaka K (1996) Newly identified pair of proteasomal subunits regulated reciprocally by interferon gamma. *J Exp Med* **183**: 1807–1816

Hochstrasser M (1995) Ubiquitin, proteasomes, and the regulation of intracellular protein degradation. *Curr Opin Cell Biol* **7**: 215–223

Hou D, Cenciarelli C, Jensen JP, Nguygen HB and Weissman AM (1994) Activation-dependent ubiquitination of a T cell antigen receptor subunit on multiple intracellular lysines. *J Biol Chem* **269**: 14244–14247

Hunter T and Pines J (1994) Cyclins and cancer. II. Cyclin D and CDK inhibitors come of age (see comments). *Cell* **79**: 573–582

Ikehata H, Kaneda S, Yamao F, Seno T, Ono T and Hanaoka F (1997) Incubation at the nonpermissive temperature induces deficiencies in UV resistance and mutagenesis in mouse mutant cells expressing a temperature-sensitive ubiquitin-activating enzyme (E1). *Mol Cell Biol* **17**: 1484–1489

Imajoh Ohmi S, Kawaguchi T, Sugiyama S, Tanaka K, Omura S and Kikuchi H (1995) Lactacystin, a specific inhibitor of the proteasome, induces apoptosis in human monoblast U937 cells. *Biochem Biophys Res Commun* **217**: 1070–1077

Jariel Encontre I, Pariat M, Martin F, Carillo S, Salvat C and Piechaczyk M (1995) Ubiquitinylation is not an absolute requirement for degradation of c-Jun protein by the 26S proteasome. *J Biol Chem* **270**: 11623–11627

Keyomarsi K and Pardee AB (1993) Redundant cyclin overexpression and gene amplification in breast cancer cells. *Proc Natl Acad Sci USA* **90**: 1112–1116

Keyomarsi K, O'Leary N, Molnar G, Lees E, Fingert HJ and Pardee AB (1994) Cyclin E, a potential prognostic marker for breast cancer. *Cancer Res* **54**: 380–385

Keyomarsi K, Conte D, Jr, Toyofuku W and Fox MP (1995) Deregulation of cyclin E in breast cancer. *Oncogene* **11**: 941–950

King RW (1996) How proteolysis drives the cell cycle. *Science* **274**: 1652–1659

Kuckelkorn U, Frentzel S, Kraft R, Kostka S, Groettrup M and Klbetzel PM (1995) Incorporation of major histocompatibility complex-encoded subunits LMP2 and LMP7 changes the quality of the 20S proteasome polypeptide processing products independent of interferon-gamma. *Eur J Immunol* **25**: 2605–2611

Kumada K, Yanagida M and Toda T (1996) Caffeine-resistance in fission yeast is caused by mutations in a single essential gene, crm1+. *Mol Gen Genet*, **250**: 59–68

Lam YA, Xu W, DeMartino GN and Cohen RE (1997) Editing of ubiquitin conjugates by an isopeptidase in the 26S proteasome. *Nature* **385**: 737–740

Lee HW, Smith L, Pettit GR, Vinitsky A and Smith JB (1996) Ubiquitination of protein kinase C-alpha and degradation by the proteasome. *J Biol Chem* **271**: 20973–20976

Lillington DM, MacCallum PK, Lister TA and Gibbons B (1993) Translocation t(6;9)(p23;q34) in acute myeloid leukemia without myelodysplasia or basophilia: two cases and a review of the literature. *Leukemia* **7**: 527–531

Loda M, Cukor B, Tam SW, Lavin P, Fiorentino M, Draetta GF, Jessup JM and Pagano M (1997) Increased proteasome-dependent degradation of the cyclin-dependent kinase inhibitor p27 in aggressive colorectal carcinomas. *Nature Med* **3**: 231–234

Löwe J, Stock D, Jap B, Zwickl P, Baumeister W and Huber R (1995) Crystal structure of the 20S proteasome from the Archaeon *T. acidophilum* at 3.4 Å resolution. *Science* **268**: 533–539

Maki CG, Huibregtse JM and Howley PM (1996) In vivo ubiquitination and proteasome-mediated degradation of p53. *Cancer Res* **56**: 2649–2654

Masutani C, Sugasawa K, Yanagisawa J, Sonoyama T, Ui M, Enomoto T, Takio K, Tanaka K, Van der Spek PJ, Bootsma D, Hoeijmakers JHJ and Hanaoka F (1994) Purification and cloning of a nucleotide excision repair complex involving the xeroderma pigmentosum group C protein and a human homologue of yeast RAD23. *Embo J* **13**: 1831–1843

Michalek MT, Grant EP, Gramm C, Goldberg AL and Rock KL (1993) A role for the ubiquitin-dependent proteolytic pathway in MHC class I-restricted antigen presentation. *Nature* **363**: 552–554

Moffat GJ, McLaren AW and Wolf CR (1994) Involvement of Jun and Fos proteins in regulating transcriptional activation of the human pi class glutathione S-transferase gene in multidrug-resistant MCF7 breast cancer cells. *J Biol Chem* **269**: 16397–16402

Mori S, Kanaki H, Tanaka K, Morisaki N and Saito Y (1995) Ligand-activated platelet-derived growth factor beta-receptor is degraded through proteasome-dependent proteolytic pathway. *Biochem Biophys Res Commun* **217**: 224–229

Morosetti R, Kawamata N, Gombart AF, Miller CW, Hatta Y, Hirama T, Said JW, Tomonaga M and Koeffler HP (1995) Alterations of the p27KIP1 gene in non-Hodgkin's lymphomas and adult T-cell leukemia/lymphoma. *Blood* **86**: 1924–1930

Musti AM, Treier M and Bohmann D (1997) Reduced ubiquitin-dependent degradation of c-Jun after phosphorylation by MAP kinases. *Science* **275**: 400–402

Nandi D, Jiang H and Monaco JJ (1996) Identification of MECL-1 (LMP-10) as the third IFN-gamma-inducible proteasome subunit. *J Immunol* **156**: 2361–2364

Pagano M, Tam SW, Theodoras AM, Beer Romero P, Del Sal G, Chau V, Yew PR, Draetta GF and Rolfe M (1995) Role of the ubiquitin–proteasome pathway in regulating abundance of the cyclin-dependent kinase inhibitor p27. *Science* **269**: 682–685

Pahl HL and Baeuerle PA (1996) Control of gene expression by proteolysis. *Curr Opin Cell Biol* **8**: 340–347

Palombella VJ, Rando OJ, Goldberg AL and Maniatis T (1994) The ubiquitin–proteasome pathway is required for processing the NF-kappa B1 precursor protein and the activation of NF-kappa B. *Cell* **78**: 773–785

Papa FR and Hochstrasser M (1993) The yeast DOA4 gene encodes a deubiquitinating enzyme related to a product of the human tre-2 oncogene. *Nature* **366**: 313–319

Peters JM (1994) Proteasomes: protein degradation machines of the cell. *Trends Biochem Sci* **19**: 377–382

© Cancer Research Campaign 1998

Peters JM, Cejka Z, Harris JR, Kleinschmidt JA and Baumeister W (1993) Structural features of the 26 S proteasome complex. *J Mol Biol* **234**: 932–937

Philip PA and Harris AL (1995) Potential for protein kinase C inhibitors in cancer therapy. *Cancer Treat Res* **78**: 3–27

Pinkus R, Weiner LM and Daniel V (1996) Role of oxidants and antioxidants in the induction of AP-1, NF-kappaB, and glutathione S-transferase gene expression. *J Biol Chem* **271**: 13422–13429

Polyak K (1994) p27/kip1, a cyclin-Cdk inhibitor, links transforming growth factor-beta and contact inhibition to cell cycle arrest. *Genes Dev* **8**: 9–22

Porter PL, Malone KE, Heagerty PJ, Alexander GM, Gatti LA, Firpo EJ, Daling JR and Roberts JM (1997) Expression of cell-cycle regulators p27/Kip1 and cyclin E, alone or in combination, correlate with survival in young breast cancer patients. *Nature Med* **3**: 222–225

Restifo NP, Esquivel F, Kawakami Y, Yewdell JW, Mule JJ, Rosenberg SA and Bennink JR (1993) Identification of human cancers deficient in antigen processing. *J Exp Med* **177**: 265–272

Ritke MK, Bergoltz VV, Allan WP and Yalowich JC (1994) Increased c-jun/AP-1 levels in etoposide-resistant human leukemia K562 cells. *Biochem Pharmacol* **48**: 525–533

Rock KL, Gramm C, Rothstein L, Clark K, Stein R, Dick L, Hwang D and Goldberg AL (1994) Inhibitors of the proteasome block the degradation of most cell proteins and the generation of peptides presented on MHC class I molecules. *Cell* **78**: 761–771

Rolfe M, Beer Romero P, Glass S, Eckstein J, Berdo I, Theodoras A, Pagano M and Draetta G (1995) Reconstitution of p53-ubiquitinylation reactions from purified components: the role of human ubiquitin-conjugating enzyme UBC4 and E6-associated protein (E6AP). *Proc Natl Acad Sci USA* **92**: 3264–3268

Rotem Yehudar R, Groettrup M, Soza A, Kloetzel PM and Ehrlich R (1996) LMP-associated proteolytic activities and TAP-dependent peptide transport for class 1 MHC molecules are suppressed in cell lines transformed by the highly oncogenic adenovirus 12. *J Exp Med* **183**: 499–514

Rubin D and Finley D (1995) The proteasome: a protein-degrading organelle. *Curr Biol* **5**: 854–858

Sadoul R, Fernandez PA, Quiquerez AL, Martinou I, Maki M, Schroter M, Becherer JD, Irmler M, Tschopp J and Martinou JC (1996) Involvement of the proteasome in the programmed cell death of NGF-deprived sympathetic neurons. *Embo J* **15**: 3845–3852

Scheffner M, Werness BA, Huibregtse JM, Levine AJ and Howley PM (1990) The E6 oncoprotein encoded by human papillomavirus types 16 and 18 promotes the degradation of p53. *Cell* **63**: 1129–1136

Scheffner M, Huibregtse JM, Vierstra RD and Howley PM (1993) The HPV-16 E6 and E6-AP complex functions as a ubiquitin-protein ligase in the ubiquitination of p53. *Cell* **75**: 495–505

Schreiber M, Baumann B, Cotten M, Angel P and Wagner EF (1995) Fos is an essential component of the mammalian UV response. *Embo J* **14**: 5338–5349

Seliger B, Hohne A, Knuth A, Bernhard H, Meyer T, Tampe R, Momburg F and Huber C (1996) Analysis of the major histocompatibility complex class I antigen presentation machinery in normal and malignant renal cells: evidence for deficiencies associated with transformation and progression. *Cancer Res* **56**: 1756–1760

Sepp Lorenzino L, Ma Z, Lebwohl DE, Vinitsky A and Rosen N (1995) Herbimycin A induces the 20 S proteasome- and ubiquitin-dependent degradation of receptor tyrosine kinases. *J Biol Chem* **270**: 16580–16587

Shaeffer JR and Cohen RE (1996) Differential effects of ubiquitin aldehyde on ubiquitin and ATP-dependent protein degradation. *Biochemistry* **35**: 10886–10893

Shimanuki M, Saka Y, Yanagida M and Toda T (1995) A novel essential fission yeast gene pad1+ positively regulates pap1(+)-dependent transcription and is implicated in the maintenance of chromosome structure. *J Cell Sci* **108**: 569–579

Shinohara K, Tomioka M, Nakano H, Tone S, Ito H and Kawashima S (1996) Apoptosis induction resulting from proteasome inhibition. *Biochem J* **317**: 385–388

Sibille C, Gould KG, Willard Gallo K, Thomson S, Rivett AJ, Powis S, Butcher GW and De Baetselier P (1995) LMP2+ proteasomes are required for the presentation of specific antigens to cytotoxic T lymphocytes. *Curr Biol* **5**: 923–930

Slingerland JM (1994) A novel inhibitor of cyclin/Cdk activity detected in TGF-beta arrested epithelial cells. *Mol Cell Biol* **14**: 3683–3694

Spataro V, Toda T, Craig R, Seeger M, Dubiel W, Harris AL and Norbury C (1997) Resistance to diverse drugs and to ultraviolet light conferred by overexpression of a novel human 265 proteasome subunit. *J Biol Chem* (in press)

Toda T, Shimanuki M, Saka Y, Yamano H, Adachi Y, Shirakawa M, Kyogoku Y and Yanagida M (1992) Fission yeast pap1-dependent transcription is negatively regulated by an essential nuclear protein, crm1. *Mol Cell Biol* **12**: 5474–5484

Treier M, Staszewski LM and Bohmann D (1994) Ubiquitin-dependent c-Jun degradation in vivo is mediated by the delta domain. *Cell* **78**: 787–798

Tsurumi C, Ishida N, Tamura T, Kakizuka A, Nishida E, Okumura E, Kishimoto T, Inagaki M, Okazaki K, Sagata N, Ishihara A and Tanaka K (1995*a*) Degradation of c-Fos by the 26S proteasome is accelerated by c-Jun and multiple protein kinases. *Mol Cell Biol* **15**: 5682–5687

Tsurumi C, DeMartino GN, Slaughter CA, Shimbara N and Tanaka K (1995*b*) cDNA cloning of p40, a regulatory subunit of the human 26S proteasome, and a homolog of the Mov-34 gene product. *Biochem Biophys Res Commun* **210**: 600–608

Van Antwerp DJ, Martin SJ, Kafri T, Green D and Verma IM (1996) Suppression of TNF-alfa-induced apoptosis by NF-κB. *Science* **274**: 787–789

Wang CY, Mayo MW and Baldwin ASJ (1996) TNF- and cancer therapy-induced apoptosis: potentiation by inhibition of NF-κB. *Science* **274**: 784–787

Watkins JF, Sung P, Prakash L and Prakash S (1993) The *Saccharomyces cerevisiae* DNA repair gene RAD23 encodes a nuclear protein containing a ubiquitin-like domain for biological function. *Mol Cell Biol* **13**: 7757–7765

Yao KS, Godwin AK, Johnson SW, Ozols RF, O'Dwyer PJ and Hamilton TC (1995) Evidence for altered regulation of gamma-glutamylcysteine synthetase gene expression among cisplatin-sensitive and cisplatin-resistant human ovarian cancer cell lines. *Cancer Res* **55**: 4367–4374

Zhou M, Wu X and Ginsberg HN (1996) Evidence that a rapidly turning over protein, normally degraded by proteasomes, regulates hsp72 gene transcription in HepG2 cells. *J Biol Chem* **271**: 24769–24775

© Cancer Research Campaign 1998